# A neuraminidase-targeting nanobody as a therapeutic candidate against influenza A and B viruses

**DOI:** 10.1128/jvi.00874-26

**Published:** 2026-08-31

**Authors:** Donglan Liu, Yuxuan Zhang, Min Zhang, Raoqing Guo, Wanwan Peng, Ren Cao, Xiaoyun Yang, Jiayan Chen, Si Huang, Jinwei Li, Chen Sheng, Liuxing Qin

**Affiliations:** 1 State Key Laboratory of Respiratory Disease, National Clinical Research Center for Respiratory Disease, Guangzhou Institute of Respiratory Health, the First Affiliated Hospital of Guangzhou Medical University 664066, Guangzhou, China; 2 Guangzhou National Laboratory, Bio-island 612039, Guangzhou, China; 3 Guangzhou Medical University 26468, Guangzhou, China; 4 School of Chemistry and Chemical Engineering, University of South China 34706 , Hengyang, China; University of North Carolina at Chapel Hill, Chapel Hill, North Carolina, USA

**Keywords:** influenza virus, neuraminidase, nanobody, antiviral mechanisms, immunotherapy

## Abstract

Influenza viruses continue to pose a significant threat to human and animal health. However, the limited number of licensed antivirals is increasingly compromised by drug resistance driven by high mutation rates. This highlights the urgent need for broad-spectrum therapeutics with novel mechanisms of action. Nanobodies, a new generation of antibody drugs, have great potential in the treatment of influenza virus infections. Neuraminidase (NA) mediates the sialic acid cleavage required for viral release, and its genetic drift is generally slower than that of hemagglutinin (HA) in influenza viruses, making it an attractive target for broad-spectrum antiviral and vaccine development. To construct a phage display nanobody library targeting NA, an alpaca was immunized with the NA protein of the H9N2 influenza virus. A specific nanobody, designated F4, was subsequently screened from the immune library. The nanobody was engineered into an Fc-fused nanobody, F4-Fc, which exhibited inhibitory activity against multiple influenza A and B viruses *in vitro* and provided robust prophylactic and therapeutic protection against influenza A and influenza B virus infections *in vivo*. Mechanistically, F4-Fc inhibits NA enzymatic activity and mediates antibody-dependent cellular cytotoxicity. In conclusion, F4-Fc demonstrates prophylactic and therapeutic efficacy against influenza A and B viruses, representing a promising antiviral drug candidate for influenza virus infection.

**IMPORTANCE:**

Influenza viruses seriously threaten human and animal health, and drugs are crucial for controlling influenza outbreaks. However, the limited variety of existing anti-influenza virus medicines and the high mutation rate of the virus have led to the continuous emergence of drug-resistant strains. Neuraminidase (NA) is a critical surface glycoprotein that exhibits slower antigenic drift than hemagglutinin (HA), making it an attractive target for cross-protective antiviral development. However, broadly active NA-targeting nanobodies, particularly those effective against both influenza A and B viruses, remain limited. Here, we constructed an Fc-fused F4 nanobody (F4-Fc) targeting neuraminidases from multiple influenza A and B viruses and demonstrated its antiviral efficacy *in vitro* and protective activity *in vivo*, highlighting its potential as a promising strategy for the prevention and treatment of influenza virus infection.

## INTRODUCTION

Seasonal influenza is one of the leading causes of severe respiratory illness, accounting for an estimated 300,000 to over 600,000 deaths worldwide annually. Influenza viruses infect a wide range of hosts, including humans, pigs, bats, and birds (1). As a zoonosis, influenza poses a dual threat to public health and the livestock industry, causing not only significant economic losses but also serious public health concerns. Notably, all four influenza pandemics in the past century have been associated with animal-origin influenza viruses (2). Additionally, ecological studies of influenza viruses have suggested that all mammalian influenza viruses ultimately derive from avian influenza viruses (3). Among avian influenza viruses, the low-pathogenicity H9N2 subtype poses a significant threat to poultry production. Importantly, H9N2 serves as a gene donor and undergoes gene exchange with viruses such as H7N9, H10N8, H5N2, and H5N6, resulting in the generation of new strains with pandemic potential (4, 5). Although hemagglutinin (HA)-based vaccines play a key role in prevention and control, the high mutation rate of the HA gene hampers their effectiveness against rapidly evolving strains. Consequently, H9N2 remains endemic in China and continues to cause sporadic cross-species transmission. Compared with that of the HA gene, the evolution of neuraminidase (NA) is relatively conservative, making NA an increasingly attractive target for the development of next-generation influenza vaccines and antivirals (6). However, vaccination requires time to elicit protective immunity and depends on the host’s immune responsiveness. In contrast, passive immunization provides immediate protection, independent of immune status, and represents an important strategy for protecting immunocompromised individuals against viral infections (7).

HA and NA are two important viral glycoproteins on the surface of influenza virus particles that play indispensable roles in the viral life cycle (8). HA mediates viral attachment and membrane fusion, facilitating entry into host epithelial cells (9). In contrast, the primary function of NA is to release progeny virus from infected cells. This is achieved by cleaving the glycosidic bond between the HA protein on the surface of the progeny virus and the sialic acid receptor on the host cell surface. This cleavage allows the virus to spread and infect surrounding cells (10, 11). NA inhibitors exert their antiviral effects mainly by blocking this release process (12). Influenza A virus (IAV) NAs are classified into 12 subtypes, grouped into Group 1 (N1, N4, N5, and N8) and Group 2 (N2, N3, N6, N7, and N9) according to the phylogenetic tree, whereas influenza B virus (IBV) NAs constitute a separate evolutionary lineage (13). In addition, the bat-derived N10, N11, and N12 are not classified into either group (14, 15). NA is a type II transmembrane protein that forms homotetramers on the surface of influenza virions. It possesses enzymatic activity, and its cleavage function depends on this activity (16). Unlike the NA tetramer, the NA monomer or dimer has very low enzymatic activity (17). The tetrameric conformation of NA is critical for its enzymatic activity and for the induction of protective antibodies, making it an important consideration in the design and preparation of NA immunogens (18).

The influenza drugs currently used in the clinic are chemical small molecules, which have led to the emergence of drug-resistant or reduced-susceptibility strains of influenza virus due to their single target and prolonged use (19–23). Therefore, there is an urgent need to develop various forms of influenza drugs with novel antiviral mechanisms to cope with the threat of drug-resistant strains. Antibody-based antiviral therapeutics, including monoclonal antibodies and nanobodies, show great potential and are considered attractive therapeutic directions for the future. Nanobodies are a unique class of single-domain antibodies derived from the variable domain (VHH) of heavy-chain-only antibodies (HcAbs) found in camelids and sharks. Unlike conventional antibodies, HcAbs lack light chains and the CH1 domain and are composed solely of VHH, CH2, and CH3 domains (24). The VHH domain (~12 kDa–15 kDa), also referred to as a single-domain antibody or nanobody, retains full antigen-binding capacity and robust structural stability, offering a 10th of the molecular weight of IgG molecules while maintaining comparable affinity and specificity (25). Owing to their small size, high solubility, thermal and chemical stability, deep tissue penetration, ease of large-scale production, and amenability to genetic engineering, nanobodies are particularly well suited for the development of inhalable formulations and long-acting antiviral agents (26). Furthermore, the low immunogenicity of nanobodies further enhances their potential for clinical applications. Collectively, these advantages make nanobodies attractive candidates for both prophylactic and therapeutic applications against respiratory viral pathogens, including influenza viruses.

To date, most studies on anti-influenza nanobodies have focused predominantly on HA and M2 proteins as targets, whereas investigations into NA-targeting nanobodies remain limited. First, the understanding of the *in vitro* and *in vivo* antiviral effects and antiviral spectrum of NA nanobodies needs to be improved. In 2013, Harmsen et al. first reported NA-targeted nanobodies, most of which were specific to a single influenza virus NA subtype. Among them, only the nanobody IV512F cross-bound to multiple NA subtypes, except for N4. However, the antiviral activity of these NA nanobodies has not been evaluated (27). Subsequently, Cardoso et al. constructed a phage display library derived from alpacas immunized with recombinant NA protein and identified two nanobodies, N1-3-VHHm and N1-5-VHHm, both of which confer antiviral protection against the H5N1 virus both *in vitro* and *in vivo*. Among these nanobodies, N1-3-VHHm effectively protected mice from infection with oseltamivir-resistant H5N1 H274Y variants, whereas N1-5-VHHm had no such protective effect (28). Compared with previously reported nanobodies that target IAV NA, those targeting IBV NA remain largely underexplored. Recently, IBV NA-targeting nanobodies have been reported to inhibit enzymatic activity, including that of drug-resistant strains, and to provide *in vivo* protection (29). Those findings demonstrate the potential of nanobodies in the control of both IAV and IBV infections. Second, the antiviral mechanism of NA nanobodies requires further elucidation. NA-specific antibodies can exert protective effects through multiple mechanisms. They can block viral release from mucins, thereby restricting viral access to host cell surfaces. They have also been shown to interfere with viral attachment to sialic acid receptors, which is similar to the mechanism of HA-targeted antibodies, and to prevent the egress of newly produced virions from infected cells (30, 31). In addition, interactions between the antibody Fc domain and Fcγ receptors (FcγRs) on immune effector cells can mediate Fc-dependent effector functions, such as antibody-dependent cellular cytotoxicity (ADCC) and antibody-dependent cellular phagocytosis (ADCP), thereby contributing to viral control and the clearance of infected cells (32, 33). Recently, an increasing number of monoclonal antibodies targeting NA have been characterized, and their epitopes have been identified (31, 34, 35). Meanwhile, the role of NA in influenza virus immunoprotection has been gradually recognized, and the number of related studies is increasing (6). For example, a broadly neutralizing human monoclonal antibody from an H3N2-infected donor that targets influenza A and B neuraminidases has been reported, and both prophylactic and therapeutic administration protected mice from multiple different influenza virus infections (34). Similarly, monoclonal antibodies isolated from influenza B virus-infected individuals have demonstrated enzymatic inhibition and protective efficacy *in vivo* when administered prophylactically or therapeutically (36). Recent studies have shown that antibodies of NA binding to conserved sites on the underside of the NA globular head, which broadly inhibit infection by human H3N2 strains, porcine H3N2 mutant strains, and H2N2 strains, have effective prophylactic and therapeutic efficacy in a mouse model (35). These findings suggest that antibodies targeting NA have broad-spectrum binding ability and effective antiviral effects, demonstrating potential for future application in clinical therapy. To date, research on NA-targeting nanobodies remains limited, and no nanobodies that broadly inhibit influenza viruses with different NA subtypes have been reported. Therefore, developing broad NA-targeted nanobodies remains a key focus in current influenza research. Moreover, their antiviral mechanisms and potential applications require further investigation.

In this study, the N2 recombinant protein derived from H9N2 was expressed for the immunization of alpaca. Through phage display screening, we identified a nanobody, F4, that binds N2 and competes with 1G01, a previously reported human monoclonal antibody broadly inhibiting IAVs of both group 1 and group 2, as well as influenza B viruses by targeting the NA active site (34). Functional analyses demonstrated that F4-Fc exhibits inhibitory activity against multiple influenza A and B virus strains *in vitro* and *in vivo*. Mechanistically, the nanobody inhibits NA enzymatic activity and induces ADCC responses. These findings provide a promising NA-targeted nanobody candidate for influenza immunotherapy.

## RESULTS

### Immunization with a recombinant NA protein and screening of NA-targeting of H9N2 nanobodies

**Fig 1:**
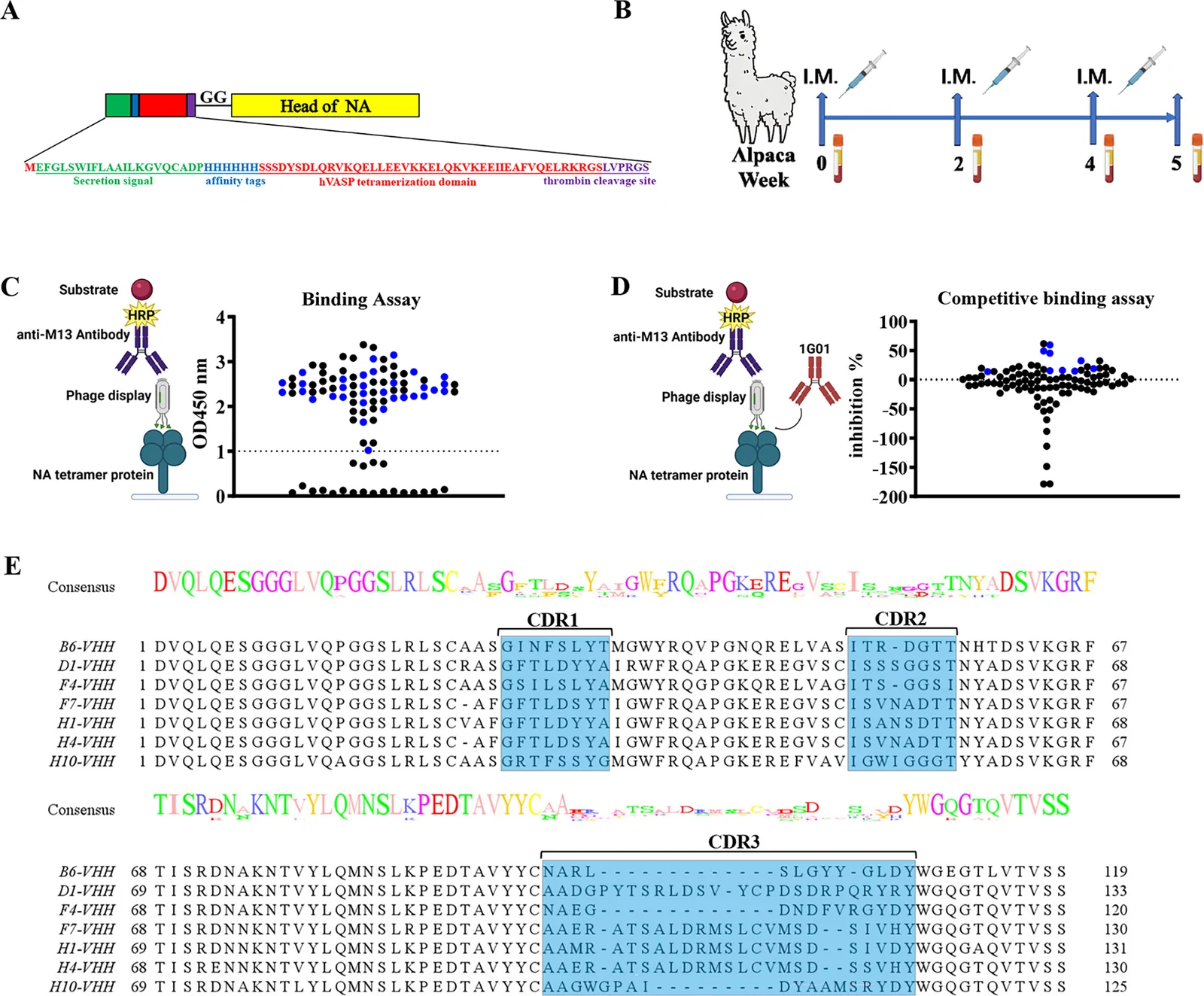
Screening of N2 nanobodies targeting H9N2. (**A**) Schematic of the recombinant NA protein. From 5′ to 3′, it encodes a secretion signal, an affinity tag, the tetramerization domain of hVASP, a thrombin cleavage site, a GC linker, and the head of NA. (**B**) Immunization timeline for alpacas with the recombinant N2 protein. Immunizations were administered on days 0, 14, and 28. Blood was collected on days 0, 14, 28, and 35 for assessment of antibody titers. PBMCs were isolated on day 35 to construct a phage-display nanobody library. (**C**) Binding ELISA to identify N2-specific nanobodies. Monoclonal clones that displayed phages were cultured and tested by ELISA using plates coated with 100 nM N2 after three rounds of panning. The OD450 was measured, and wells without phages served as negative controls (NCs). The image was created with BioRender. (**D**) Competitive ELISA was used to screen for nanobodies competing with 1G01. Monoclonal clones that displayed phages were tested by competitive ELISA, with 100 μL of 10 μg/mL 1G01 added to N2-coated plates before phage incubation. OD450 was measured, and results were compared with the results of the binding ELISA. The image was created with BioRender. (**E**) Alignment of amino acid sequences of seven isolated nanobodies generated using Jalview (version 2). The three complementarity-determining regions (CDRs) are indicated by blue boxes.

The recombinant NA protein derived from H9N2 was expressed and purified using a previously described recombinant protein design (37), in which the head of NA was genetically fused to the tetramerization domain of human vasodilator-stimulated phosphoprotein (hVASP) (Fig. 1A). The protein was secreted from mammalian cells for expression and obtained by affinity purification (Fig. S1). To verify whether the purified recombinant protein contained correctly folded tetrameric neuraminidase, NA enzymatic activity was measured using the NA-Star assay. The recombinant NA protein from the H9N2 subtype exhibited enzymatic activity, which was higher than that of other subtypes, suggesting that the purified preparation contains tetrameric neuraminidase as the enzymatically active species (Fig. S2). Purified NA protein was prepared and subsequently used for alpaca immunization. The phage-displayed VHH library was constructed from peripheral blood mononuclear cells (PBMCs) isolated 7 days after the final immunization (Fig. 1B). Using binding enzyme-linked immunosorbent assay (ELISA), we initially identified 76 N2-binding clones. Following sequencing and complementarity-determining region 3 (CDR3) analysis, clones sharing identical CDR3 sequences were clustered, and a representative clone was retained from each cluster, resulting in 32 unique N2-specific clones (Fig. 1C and Table S1). To identify cross-reactive nanobodies, these candidates were further evaluated for binding to neuraminidase proteins derived from H1N1, H3N2, H5N6, H7N9, and H9N2 influenza subtypes, among which 19 clones exhibited cross-reactive binding activity. In parallel, competitive ELISA was performed to screen nanobodies competing with mAb 1G01 for N2 binding (Fig. 1D). This analysis identified nine unique clones with relatively strong competitive activity, with D1, F7, and F4 showing the strongest competition against 1G01 (Fig. 1D and Table S2). Integrating the results from binding and competitive ELISA assays, seven nanobodies (B6, D1, F4, F7, H1, H4, and H10) were selected for further study. Sequencing analysis of individual nanobody cDNAs revealed highly diverse CDR regions among these clones (Fig. 1E).

### Characterization and antiviral evaluation of Fc-fused nanobodies

**Fig 2:**
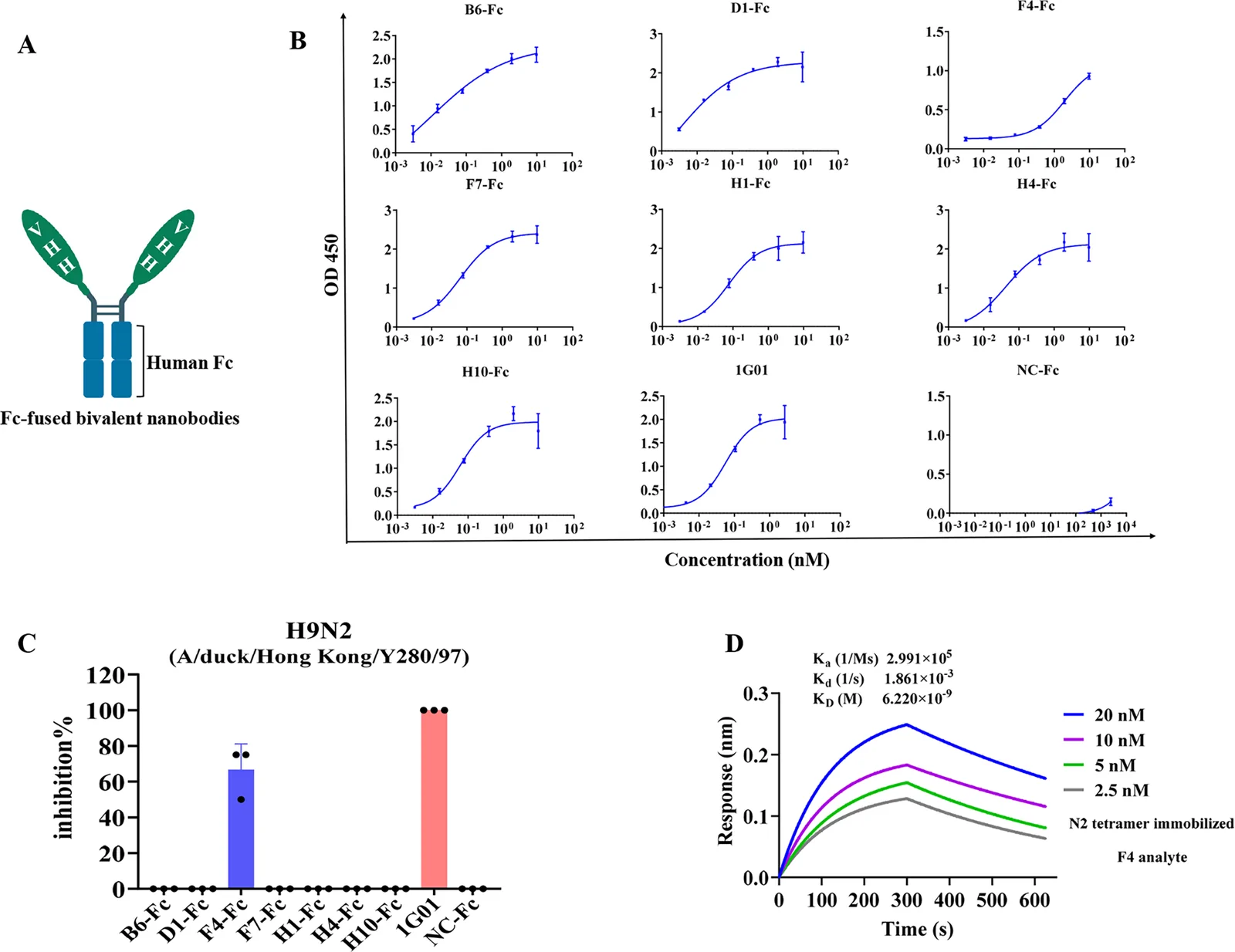
Fc-fused nanobodies bind to H9N2 NA and inhibit H9N2 virus *in vitro*. (**A**) Schematic representation of the Fc-fused nanobody construct. The nanobody VHH domain was fused to the Fc region of human IgG1 and expressed in FreeStyle 293-F (293F) cells with an N-terminal secretion signal. The image was created with BioRender. (**B**) Determination of the EC50 for F4-Fc binding to N2. ELISA plates were coated with 100 nM recombinant N2 protein from H9N2, and 5-fold serial dilutions of each Nb-Fc were added. OD450 values were measured, and EC50 values were calculated by nonlinear regression. 1G01 was used as the positive control, and Nb-Fc was used as the negative control. (**C**) *In vitro* inhibition of H9N2 by F4-Fc. The H9N2 virus (1 × 10^3^ PFU/50 μL) was incubated with each Nb-Fc at a concentration of 25 μg/mL and used to infect MDCK monolayers. After 48 h, HA titers in the supernatant were measured by hemagglutination assay. 1G01 served as a positive control, and NC-Fc was used as a negative control. (**D**) Determination of the affinity of nanobody F4 for N2. Biotinylated N2 was immobilized on streptavidin (SA) biosensors and incubated with serial twofold dilutions (2.5 nM–20 nM) of F4 nanobody for BLI.

To increase the therapeutic potential of nanobodies, Fc-fused nanobodies (Nb-Fc) were obtained by fusing the nanobodies with the human IgG1 Fc domain (Fig. 2A). We characterized the N2-binding ability of the Nb-Fc by ELISA. All seven constructs retained binding activity to N2 after Fc fusion (Fig. 2B and Table S3). The antiviral activities of the Nb-Fc constructs were assessed *in vitro*. The results demonstrated that F4-Fc, alongside the positive control antibody 1G01, exhibited potent inhibitory activity against H9N2 virus in MDCK cells, whereas the other six Nb-Fc constructs and the negative control NC-Fc antibody showed no detectable antiviral activity (Fig. 2C). Furthermore, the affinity of the F4 nanobody for N2 was quantified by bio-layer interferometry (BLI), revealing a Kd of 6.22 nM (Fig. 2D).

### F4-Fc exhibits cross-subtype NA inhibitory activity and ADCC-mediated antiviral functions

**Fig 3:**
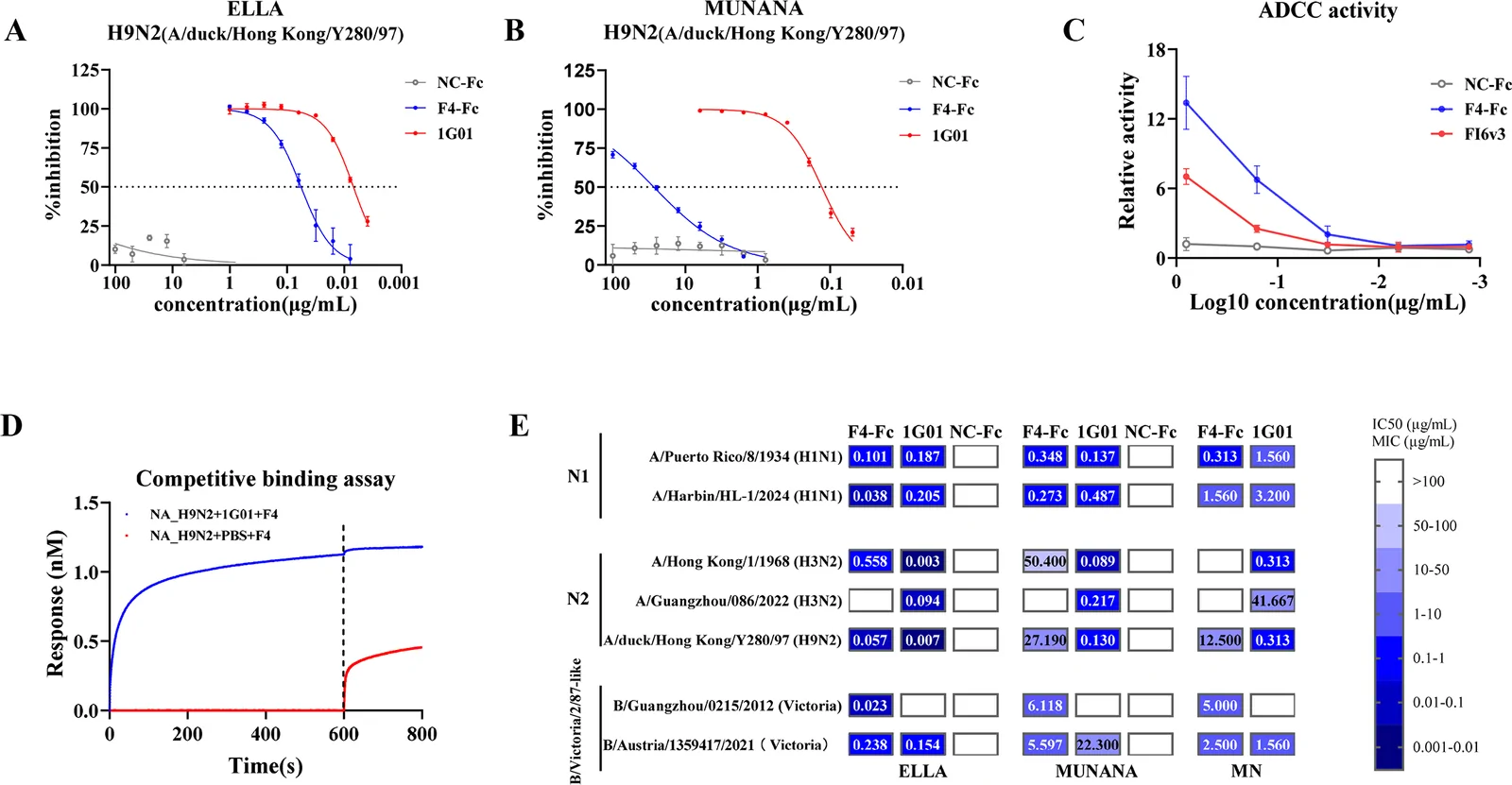
*In vitro* functional activities of F4-Fc. (**A**) NA inhibition of F4-Fc against H9N2 (A/duck/Hong Kong/Y280/97) was measured by ELLA. 1G01 served as the positive control, and the nanobody NC-Fc was used as a negative control. (**B**) NA inhibition of F4-Fc against H9N2 (A/duck/Hong Kong/Y280/97) strain was assessed by NA-Star assay. 1G01 was used as the positive control, and the nanobody NC-Fc was used as a negative control. (**C**) Detection of the ADCC induced by F4-Fc. The HA antibody FI6V3, which is known to induce ADCC, was used as a positive control. A non-binding nanobody Fc fusion construct NC-Fc was used as a negative control. (**D**) Competitive binding between F4 and 1G01 for N2 was assessed by BLI. Biotinylated N2 was immobilized on SA biosensors, pre-incubated with either 1G01 or PBS (negative control), and subsequently subjected to F4 binding analysis. (**E**) Heat map of antibody NA inhibition activity in the ELLA and MUNANA assays and virus inhibition in the MN assay. Half maximal inhibitory concentration (IC50) values of F4-Fc and 1G01 against NAs were using ELLA and MUNANA assay. Determination of the MIC of F4-Fc against influenza viruses by MN assay. The viruses were mixed with serial twofold dilutions of antibodies and added to MDCK cells for 48 h, respectively. Hemagglutination assay was used to measure HA titers in the supernatants. The lowest antibody concentration that completely prevented detectable hemagglutination was defined as the MIC, and the 1G01 antibody served as a positive control. NC-Fc served as the negative control.

To reveal the mechanism by which the F4-Fc antibody exerts its inhibitory effect, ELLA (enzyme-linked lectin assay) and MUNANA assay were used to detect the inhibitory activity of F4-Fc against N2. Both methods demonstrated that F4-Fc effectively inhibited N2 enzymatic activity with half-maximal inhibitory concentration (IC50) values of 0.05663 μg/mL and 27.19 μg/mL, respectively (Fig. 3A and B). Previous studies have reported that ADCC is also one of the main antiviral mechanisms mediated by anti-NA antibodies (38). Here, the ability of F4-Fc to activate ADCC was assessed using a luciferase reporter assay, which demonstrated that the F4-Fc antibody could induce ADCC (Fig. 3C). Previous studies have demonstrated that the human-derived monoclonal antibody 1G01 possesses broad-spectrum anti-influenza virus activity (33). To determine whether the F4 nanobody identified in this study has similar cross-reactive potential, we assessed its ability to compete with 1G01 for N2 binding using BLI. F4 effectively competed with 1G01 (Fig. 3D), indicating that the two antibodies recognized overlapping epitopes on N2 and suggesting that F4 may share a similar antiviral spectrum. ELLA and MUNANA assay were employed to evaluate the NAI activity of F4-Fc; 1G01 and NC-Fc were used as the reference antibodies. Both F4-Fc and 1G01 displayed NAI activity against multiple influenza A N1, N2, and influenza B/Victoria/2/87-like viruses, with 1G01 and Nb-Fc serving as positive and negative controls, respectively (Table S4). The results showed that F4-Fc exhibited inhibitory activity against several N1, N2, and B/Victoria/2/87-like lineage influenza viruses to varying degrees, whereas the negative control Nb-Fc showed no detectable inhibitory activity in any of the assays. Among N1 subtype viruses, F4-Fc demonstrated effective NA inhibitory activity. Against A/Puerto Rico/8/1934 (H1N1) and A/Harbin/HL-1/2024 (H1N1), F4-Fc exhibited stronger inhibitory activity than 1G01 in the ELLA. Notably, against the recent isolate A/Harbin/HL-1/2024, F4-Fc showed superior inhibitory activity to 1G01 in the ELLA, MUNANA, and microneutralization (MN) assay, indicating that F4-Fc possesses potent inhibitory activity against N1 subtype viruses. For N2 subtype viruses, the inhibitory activity of F4-Fc varied among different strains. F4-Fc retained detectable inhibitory activity against A/Hong Kong/1/1968 (H3N2) and A/duck/Hong Kong/Y280/97 (H9N2) in the ELLA; however, its overall inhibitory potency was lower than that of 1G01, particularly in the MUNANA and MN assays. In addition, F4-Fc showed no detectable inhibitory activity against A/Guangzhou/086/2022 (H3N2) (IC50 >100 µg/mL), suggesting limited coverage against certain N2 viruses. In contrast, 1G01 maintained potent inhibitory activity against all tested N2 viruses. Notably, F4-Fc exhibited strong inhibitory activity against B/Victoria/2/87-like lineage viruses. Against B/Guangzhou/0215/2012 (Victoria), F4-Fc showed robust inhibitory activity in the ELLA, MUNANA, and MN assays, whereas 1G01 showed no detectable activity in any of the three assays (IC50 >100 µg/mL), indicating that F4-Fc displayed superior inhibitory potency against this strain. Furthermore, F4-Fc retained inhibitory activity against another B/Victoria/2/87-like strain, B/Austria/1359417/2021, with potency comparable to that of 1G01, suggesting cross-reactive inhibitory activity against B/Victoria/2/87-like lineage viruses.

Collectively, these results demonstrate that F4-Fc possesses cross-reactive inhibitory activity against multiple influenza virus subtypes, particularly against N1 subtype and B/Victoria/2/87-like lineage viruses.

### F4-Fc provides prophylactic protection against multiple influenza virus subtypes

**Fig 4:**
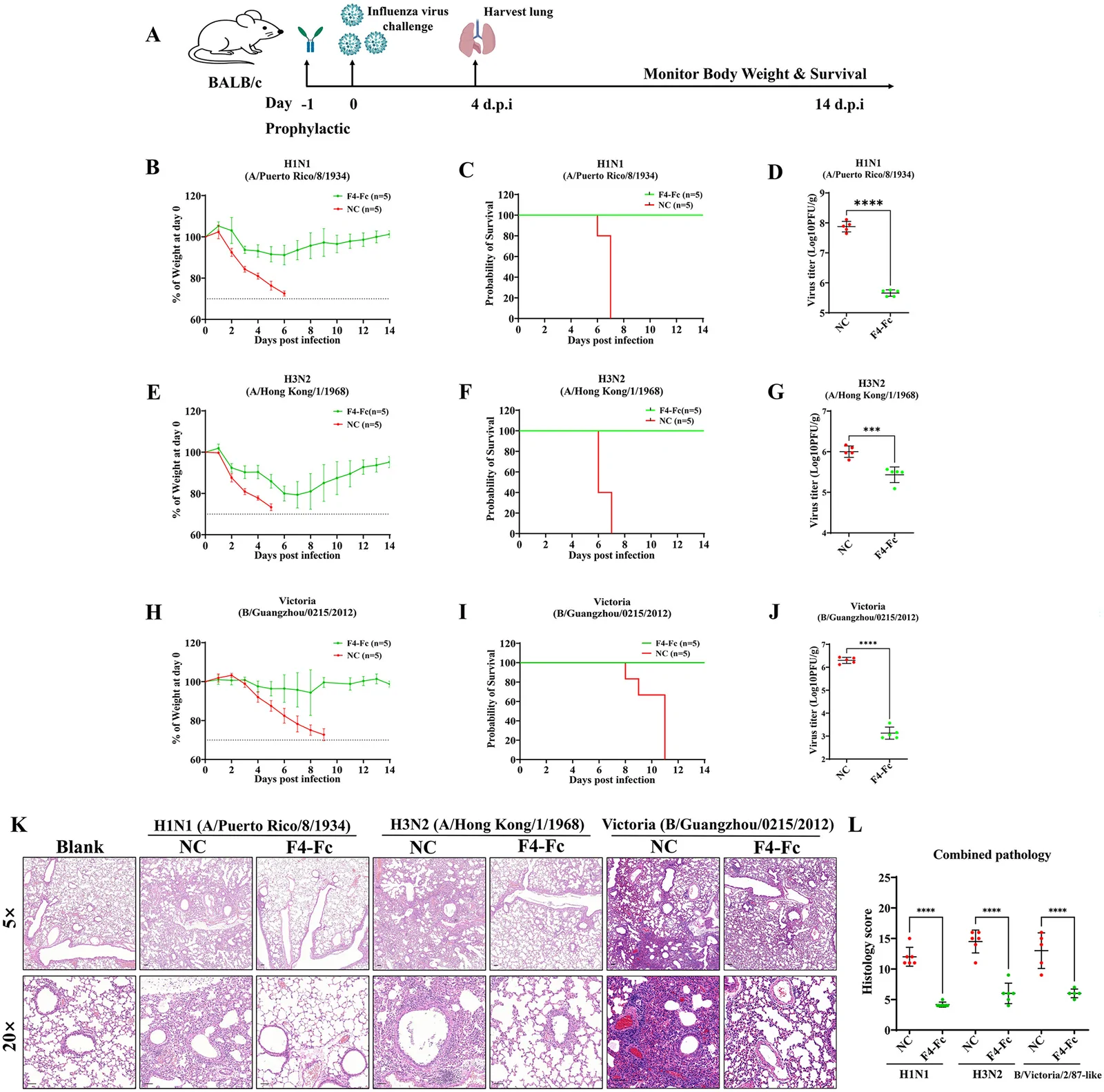
*In vivo* prophylactic protective efficacy of F4-Fc. (**A**) Schematic diagram of the *in vivo* experimental procedure for prophylactic studies. 7-week-old female BALB/c mice (*n* = 5) were used to evaluate *in vivo* prophylactic protection of F4-Fc. F4-Fc was administered intraperitoneally before viral challenge. PBS-treated, Nb-Fc-treated, and untreated groups served as controls. The mice were intranasally challenged with H1N1 (A/Puerto Rico/8/1934), H3N2 (A/Hong Kong/1/1968), or Victoria (B/Guangzhou/0215/2012) 24 h after treatment. The image was created with BioRender. (**B**, **C**, **E**, **F**, **H**, and **I**) Percent body weight changes and survival rates were monitored for 14 days post-infection. The humane endpoint was defined as 30% weight loss from day 0, as indicated by the dotted line. (**D**, **G**, and** J**) Determination of viral titers in lung tissues. At 4 days after virus infection, lung tissues were collected to determine viral titers using PFA. (**K**) Histological analysis of lung tissues stained with H&E. Histological evaluation was performed on H&E-stained lung sections (*n* = 5 mice per group), and one representative image is presented. Scale bar = 100 nm (**L**) Blinded scoring of combined pathology. Pulmonary inflammation was assessed on the basis of four parameters: peribronchiolitis, perivasculitis, alveolitis, and interstitial pneumonia.

To further evaluate the prophylactic protective capacity of F4-Fc *in vivo*, 7-week-old female BALB/c mice were intraperitoneally administered 5 mg/kg F4-Fc for 24 h before intranasal challenge with influenza viruses. PBS- or Nb-Fc-treated mice were used as NCs. A subset of the mice was sacrificed at 4 days post-infection (dpi) to assess the viral load and pathological changes in the lung tissues. The remaining mice were monitored daily for body weight and survival until 14 days post-infection (Fig. 4A). In the A/Puerto Rico/8/1934 (H1N1) infection model, viral titers in lung homogenates were quantified using a plaque-forming assay (PFA) at 4 days post-infection. Administration of F4-Fc resulted in an approximately 165-fold reduction in pulmonary viral titers compared to the NC group (Fig. 4D). F4-Fc conferred complete protection against lethal challenge with A/Puerto Rico/8/1934 (H1N1) (Fig. 4C), with all the mice surviving and their body weight loss remaining below 10%. Moreover, the mice recovered to their initial body weight and continued to gain weight during the observation period (Fig. 4B). In the A/Hong Kong/1/1968 (H3N2) challenge model, viral titers in lung homogenates were also detected at 4 dpi, showing an approximately fourfold reduction in viral titers with F4-Fc treatment compared to the NC group (Fig. 4G). Notably, although some weight loss occurred, all mice eventually recovered to their original weight and continued to gain weight during the observation period (Fig. 4E). Importantly, F4-Fc provided complete protection in the mouse-lethal model (Fig. 4F). Similarly, in the B/Guangzhou/0215/2012 (Victoria) challenge model, viral titers in lung homogenates were further determined at 4 dpi, F4-Fc treatment markedly reduced pulmonary viral loads, with an approximately 1,479-fold reduction compared with the NC group (Fig. 4J). Throughout the infection period, F4-Fc-treated mice exhibited mild body weight loss and were completely protected from lethal viral challenge (Fig. 4H and I). Lung tissues were stained with hematoxylin and eosin (H&E) and examined for histological changes in blood vessels, bronchioles, terminal bronchioles, and alveolar lumens. Compared with the NC group, F4-Fc treatment effectively reduced pulmonary inflammation, alleviating peribronchiolitis, perivasculitis, alveolitis, and interstitial pneumonia (Fig. 4K and L). Together, these results demonstrate that F4-Fc provides potent prophylactic protection against lethal influenza virus infection by effectively suppressing viral titers and alleviating lung pathology *in vivo*.

### F4-Fc provides therapeutic protection against multiple influenza virus subtypes

**Fig 5:**
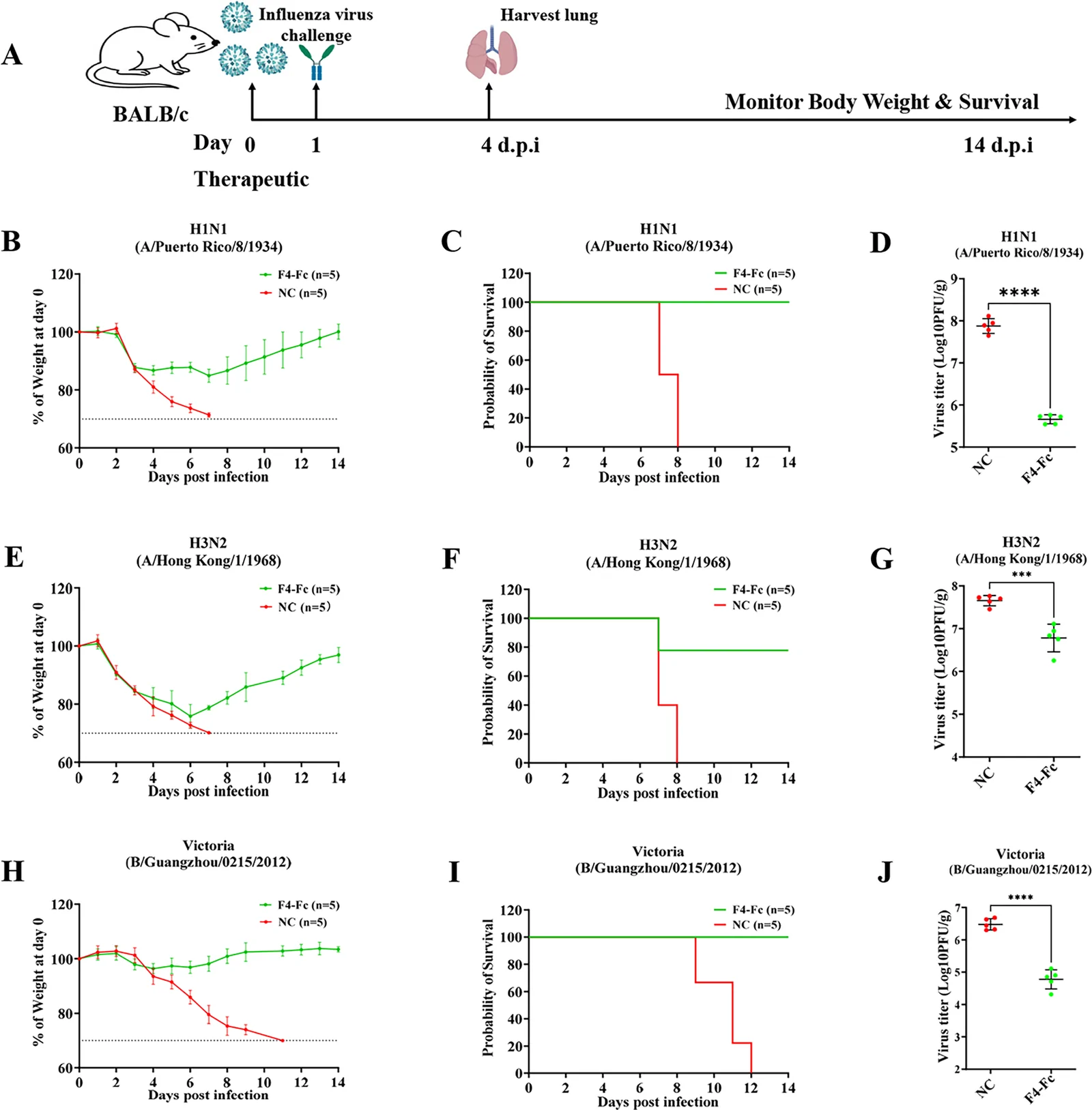
*In vivo* therapeutic protective efficacy of F4-Fc. (**A**) Schematic of the *in vivo* therapeutic protection experimental procedure. 7-week-old female BALB/c mice were used to evaluate the *in vivo* therapeutic protective activity of the antibodies, with five mice per group (*n* = 5). 24 h before antibody administration, the mice were intranasally infected with H1N1(A/Puerto Rico/8/1934), H3N2 (A/Hong Kong/1/1968), or B/Victoria (B/Guangzhou/0215/2012). 24 h post-infection, the F4-Fc antibody was intraperitoneally injected at a dose of 5 mg/kg, whereas the negative control group (NC) received an equal volume of a negative control antibody. (**B**, **C**, **E**, **F**, **H**, and** I**) Percentage changes in body weight and survival rate of mice. The percentage change in body weight and survival rate of the mice was monitored within 14 days post-infection. The humane endpoint was defined as a 30% reduction in the initial body weight, as indicated by the dotted line. (**D**, **G**, and **J**) Quantification of pulmonary viral load. Lungs were harvested at 4 dpi, and viral titers were determined by PFA.

To evaluate the therapeutic efficacy of F4-Fc in lethal influenza virus infection models, mice were intranasally challenged with different influenza virus subtypes, followed by intraperitoneal administration of 5 mg/kg F4-Fc or an equal volume of negative control antibody at 24 h post-infection. Body weight and survival were monitored daily for 14 days (Fig. 5A). Compared with the NC group, F4-Fc treatment alleviated body weight loss in mice infected with A/Puerto Rico/8/1934 (H1N1), A/Hong Kong/1/1968 (H3N2), and B/Guangzhou/0215/2012 (Victoria lineage). Moreover, surviving mice in the F4-Fc-treated groups gradually recovered to their initial body weight by 14 dpi (Fig. 5B, E, and H). In terms of survival, all mice in the NC group succumbed to viral infection, whereas F4-Fc treatment provided complete protection against lethal challenge with A/Puerto Rico/8/1934 (H1N1) and B/Guangzhou/0215/2012 (Victoria lineage) viruses (Fig. 5C and F). In the H3N2 challenge model, F4-Fc conferred partial protection, with four out of five treated mice surviving until the end of the observation period (Fig. 5I). Furthermore, F4-Fc treatment effectively reduced pulmonary viral loads across all infection models compared with the NC group. Specifically, lung viral titers were reduced by approximately 167-fold in the A/Puerto Rico/8/1934 (H1N1) model, 7-fold in the A/Hong Kong/1/1968 (H3N2) model, and 50-fold in the B/Guangzhou/0215/2012 (Victoria) model. These findings demonstrate that F4-Fc exhibits potent *in vivo* antiviral activity against multiple influenza virus subtypes. Collectively, these results indicate that F4-Fc provides therapeutic protection in multiple lethal influenza virus infection models by significantly reducing pulmonary viral loads, alleviating infection-associated body weight loss, and improving survival rates. Notably, F4-Fc conferred complete protection against H1N1 and B/Victoria/2/87-like virus infection and partial protection against H3N2 virus infection, highlighting its cross-subtype antiviral activity and potential therapeutic value.

## DISCUSSION

The development of effective therapeutic antibodies has emerged as a highly promising strategy for combating respiratory viral infections. For example, nirsevimab has become the first long-acting monoclonal antibody approved globally for the prevention of respiratory syncytial virus infection in infants (39). Antibody-based therapies can provide potent and specific antiviral activity against viral pathogens, making them an important complement to vaccines and antiviral drugs. As next-generation antibodies, nanobodies are receiving increasing interest for their antiviral potential. Importantly, nanobodies possess several advantages, including the ability to be readily engineered into multivalent and modular constructs, improved biochemical stability, and high levels of recombinant expression (26, 40). Designing antibodies with multispecificity capable of recognizing distinct epitopes or viral targets offers a robust approach to counteract viral escape mechanisms and maintain neutralization efficacy against evolving strains (41). In this study, the nanobody F4 displayed relatively weak inhibitory activity against N2-subtype viruses. Beyond affinity maturation strategies, future work may focus on engineering a bispecific antibody by linking F4 with another antibody exhibiting broad inhibitory activity against N2 viruses. This strategy could potentially expand the breadth of antiviral coverage and lead to the development of a broadly protective antibody against circulating seasonal influenza viruses.

Although there are approved vaccines against H9N2, H9N2 has replaced H5N6, and H7N9 has become the predominant subtype of avian influenza A virus circulating in chickens and ducks, and continues to pose a significant threat to the poultry industry (5, 42, 43). Moreover, H9N2 has attracted increasing attention due to its capacity for cross-species transmission and its association with several emerging human-infected avian influenza viruses, including H3N8, H7N9, and H10N8, for which it can serve as genetic donors to other influenza viruses by providing gene segments that undergo reassortment with those of co-circulating subtypes (4, 5). The underlying reasons for the continued circulation and evolution of H9N2 in vaccinated poultry populations remain unclear, highlighting the urgent need for novel antiviral strategies. Moreover, F4-Fc exhibited inhibitory activity *in vitro* against a swine-origin H1N1 virus (Fig. S3). Taken together, these findings suggest that F4-Fc has cross-species antiviral activity against influenza viruses.

Herein, an alpaca was immunized with the NA of H9N2, and a phage display nanobody library was constructed. The nanobody F4, which targets N2, was isolated from this library and engineered into an Fc-fused nanobody through fusion with the human IgG1 Fc (Fig. 2A). F4-Fc displayed inhibitory activity against homologous H9N2 virus and cross-inhibited heterologous subtypes *in vitro* (Fig. 3E). In prophylactic studies, F4-Fc significantly attenuated body weight loss, reduced viral titers in the lungs, and mitigated lung pathology in lethal mouse models of influenza virus infection, providing complete prophylactic protection against both H1N1, H3N2, and B/Victoria/2/87-like lineage viruses (Fig. 4). In therapeutic studies, F4-Fc conferred complete protection against H1N1 and B/Victoria/2/87-like virus infection and partial protection against H3N2 virus infection (Fig. 5). Collectively, these findings demonstrate the potential of F4-Fc as an NA-targeting therapeutic and support further development of NA-targeting nanobody-based strategies for the prevention and treatment of influenza virus infections.

In this study, both binding ELISA and competitive binding ELISA were employed for antibody screening, and the nanobody F4 that was screened in this study competed with 1G01 for binding to N2 (Fig. 3D), indicating that they share overlapping epitopes on N2. Notably, among the influenza virus strains tested in this study, F4 demonstrated inhibitory activity against B/Guangzhou/0215/2012 (Victoria), whereas 1G01 did not exhibit activity against B/Guangzhou/0215/2012 (Victoria) *in vitro*, even at the highest tested concentration (100 μg/mL). Furthermore, F4-Fc demonstrated greater inhibitory potency than 1G01 against A/Puerto Rico/8/1934 (H1N1) and A/Harbin/HL-1/2024 (H1N1) *in vitro*. In contrast, 1G01 exhibited stronger inhibitory activity against N2-subtype viruses than F4-Fc (Table S4). These findings indicate that F4 recognizes an epitope on neuraminidase that partially overlaps with that of 1G01, although the two epitopes are not identical. The precise binding site of F4 warrants further structural characterization. Our findings indicate that the antiviral activity of F4-Fc is not strictly correlated with NA subtype. Instead, it is more likely determined by the degree of conservation of the epitope recognized by F4-Fc among different influenza viruses. This interpretation is consistent with our observation that F4-Fc exhibited potent activity against certain N1 viruses but relatively limited activity against some N2 viruses, despite being originally generated against the N2 protein of an H9N2 virus. Comprehensive evaluation using a larger collection of influenza A viruses representing diverse N1 and N2 evolutionary lineages, together with epitope mapping and structural studies, will be required to fully elucidate the molecular basis underlying this differential antiviral activity.

Fc-mediated effector functions, such as ADCC, play a critical role in protection against influenza virus infection (44). NA-specific antibodies with LALA-PG mutations, which abrogate Fc effector activity, exhibit markedly reduced *in vivo* protective efficacy, highlighting the essential contribution of the Fc domain to NA antibody-mediated protection (31, 38, 45). Nanobodies lack the Fc structural domain in immunoglobulins, thereby limiting their capacity to mediate Fc-dependent effector mechanisms. Previous studies have demonstrated that the fusion of nanobodies with the Fc domain can confer Fc-mediated effector functions (46, 47). In addition to antibody engineering approaches, Fc-based conjugation strategies have also been applied to antiviral drug design. For example, CD388, a zanamivir-Fc conjugate, has been reported to enhance the pharmacokinetic properties and antiviral activity of neuraminidase inhibitors, demonstrating broad-spectrum efficacy against influenza A and B viruses in preclinical models (48). In this study, we engineered an Fc-fused nanobody F4-Fc by genetically fusing the F4 nanobody to the human IgG1 Fc domain. Subsequent functional assays using ADCC reporter cells confirmed that F4-Fc effectively mediated ADCC (Fig. 3C). These results suggest that Fc fusion is a potential strategy for enhancing the antiviral efficacy of NA-specific nanobodies.

In conclusion, the nanobody F4 was isolated from alpacas immunized with NA of H9N2 and subsequently engineered into an Fc-fused format, exhibiting activity against multiple influenza A and B viruses *in vitro* and *in vivo*. These findings underscore its potential as a therapeutic for seasonal and zoonotic influenza as well as a reserve intervention against emerging or pandemic strains.

## MATERIALS AND METHODS

### Cells, virus, and recombinant NA protein

Madin Darby canine kidney (MDCK; CCL-34, ATCC) cells were maintained in Dulbecco’s modified Eagle medium (DMEM) supplemented with 10% fetal bovine serum (Gemini), 100 U/mL penicillin, and 100 µg/mL streptomycin (Gibco). FreeStyle 293-F cells (293F; Gibco, USA) were cultured in FreeStyle 293 expression medium (Gibco, USA).

The influenza A viruses A/Puerto Rico/8/1934 (H1N1), A/Harbin/HL-1/2024 (H1N1), A/swine/China/Heilongjiang GN/2020 (H1N1), A/Hong Kong/1/1968 (H3N2), A/Guangzhou/086/2022 (H3N2), A/duck/Hong Kong/Y280/97 (H9N2), together with the influenza B viruses B/Guangzhou/0215/2012 (Victoria) and B/Austria/1359417/2021 (Victoria), were propagated in MDCK cells at 37°C (IAVs) or 35°C (IBVs) for 36–60 h. The number “97” in A/duck/Hong Kong/Y280/97 indicates the year of virus isolation (1997). All viruses were handled under biosafety level 2 conditions.

NA sequences of influenza viruses were obtained from GenBank, and the intracellular, transmembrane, and stalk domains were removed to retain only the head domain. To prepare the NA antigen for the immunization of alpaca, a plasmid expressing recombinant NA protein, encoding a secretion signal peptide, affinity purification tag, tetrameric structural domain of hVASP, prothrombin cleavage site, GC linker, and NA head region sequentially from the 5′ end was constructed. The gene fragment was synthesized and cloned into the eukaryotic expression vector pcDNA3.4 to obtain the His_NA_tetramer_pcDNA3.4 plasmid by GenScript (Nanjing, China). This plasmid was then transformed into *E. coli* DH5α. After plasmid extraction, the plasmid was transfected into 293F cells using polyethylenimine (PEI) transfection reagent. The cells were cultured in a shaker for 6 days, followed by centrifugation to collect the supernatant. The recombinant NA protein was purified by Ni-affinity chromatography.

To prepare the biotinylated NA protein, the His_NA_tetramer_AviTag_pcDNA3.4 plasmid was obtained by fusing AviTag to the C-terminal end of the His_NA_tetramer gene fragment, and the His_NA_tetramer_AviTag_pcDNA3.4 plasmid was co-transfected with the plasmid (1:1) expressing BirA in 293F cells. The cells were supplemented with biotin 4-6h after transfection. The supernatant was collected by centrifugation after 6 days of further incubation on a shaker, and the biotinylated recombinant NA protein was purified using Ni-affinity chromatography.

### Construction of phage-displayed nanobody library

Alpacas were immunized with the recombinant NA protein through one primary and two booster subcutaneous immunizations at 14 day intervals. For the primary immunization, 500 μg of NA protein was emulsified with an equal volume of complete Freund’s adjuvant (Merck KGaA, Germany). For the booster immunization, 250 μg of NA protein was emulsified with an equal volume of incomplete Freund’s adjuvant (Merck KGaA, Germany). On day 7 after the final boost, 50 mL of alpaca whole blood was collected. PBMCs were isolated and used to extract total RNA. The cDNA was obtained by reverse transcription, which was used as a template to amplify the VHH domain by PCR, and the VHH fragments were purified by agarose gel separation. The VHH fragments were cloned into the pADL-23c plasmid vector, which was subsequently transformed into *E. coli* TG1 receptor cells to construct the phage-displayed nanobody library.

### Screening of phage-displayed nanobodies that bind to NA

TG1 cells containing the VHH phage library were cultured in 200 mL of 2YT medium, and the initial OD600 value was 0.04, followed by incubation at 37°C and 250 rpm for 2 h to reach an OD600 value of 0.4. The library was inoculated with the M13 helper phage at a final concentration of 2 × 10^9^ PFU/mL, and the cells were incubated at 37°C and 250 rpm for 1 h. The cells were subsequently centrifuged at 16°C and 3500 rpm for 10 min to remove the supernatant, after which the TG1 cells were collected. TG1 cells were resuspended in 200 mL of 2YT medium and cultured overnight at 25°C and 250 rpm. The supernatant was collected by centrifugation at 4,000 rpm for 30 min at 4°C. The recombinant phage in the culture medium was collected by the polyethylene glycol precipitation method, and finally, the phage suspension was resuspended in PBS. The phage suspension was diluted 10-fold to determine the titer of the phage. To enrich for NA nanobodies, 1 × 10^12^ PFU of phage was blocked with blocking buffer and incubated at 10 rpm for 30 min at room temperature, followed by the addition of biotinylated NA antigen to the phage and incubation at 10 rpm for 1 h. The phage and biotinylated mixture were co-incubated with streptavidin magnetic beads for 7 min at 10 rpm at room temperature, and the magnetic beads were washed three times with PBS. Five hundred microliters of TBSC buffer (10 mM Tris, 137 mM NaCl, 1 mM CaCl_2_, pH 7.4) and 50 μL of 10 mg/mL trypsin were added to the magnetic beads, and the phage was digested from the magnetic beads to infect 10 mL of TG1 cells. The TG1 cells were incubated at 37°C for 30 min, 100 μL of TG1 cells was serially diluted 10-fold in 2YT medium, and 100 μL of culture from each dilution gradient was cultured on 2YT solid medium (10 cm petri dishes). The remaining culture in the tube was centrifuged at 3,500 rpm for 15 min at 16°C. The cells were resuspended in 1 mL 2YT medium and incubated on 2YT solid medium (24.5 cm petri dishes), and incubated overnight at 37°C, after which the TG1 cells were counted and collected. Three rounds of panning were carried out following the above steps.

### Screening and expression of nanobodies

Individual colonies in the 10 cm petri dishes of the final round were cultured in 96-well plates. Cultures were infected with M13 helper phage (New England Biolabs, USA) to produce recombinant phages. For ELISA, 100 μL of 100 nM recombinant NA protein was added to each well of an ELISA plate and incubated overnight at 4°C. The plates were then blocked with 3% skim milk powder in phosphate-buffered saline (MPBS). Phages were diluted in 6% MPBS (1:1) and incubated for 1 h. Bound phages were detected using horseradish peroxidase (HRP)-conjugated anti-M13 antibody (Sino Biological, China) and 3,3’,5,5′-tetramethylbenzidine substrate (KPL, USA). OD450 was measured, and wells without phage were used as negative controls. For Fc-fused nanobody production, VHHs of positive clones were fused to the human IgG1 Fc domain using PCR and cloned into the pCMV vector. The plasmids were transiently transfected into 293F cells using PEI (Polysciences, USA), and Fc-tagged nanobodies were purified from the culture supernatant.

### Microneutralization assay

MDCK cells were seeded in 96-well plates (6 × 10^3^ cells/well) for 24 h. The virus was diluted to 100 TCID50/50 μL in DMEM, and Fc-fused nanobodies were serially diluted twofold in DMEM. Equal volumes (60 μL each) of diluted virus and nanobody were mixed and incubated at 37°C for 1 h. Cells were washed with PBS, and 100 μL of the virus-antibody mixture was added to the cells. After 48 h incubation, viral titers in the supernatant of cell culture were measured using the hemagglutination assay. The lowest antibody concentration that completely prevented detectable hemagglutination was defined as the MIC value (38).

### Biolayer interferometry

Binding affinity was measured using BLI at 25°C. Biotinylated recombinant NA protein was loaded on streptavidin biosensors and equilibrated in PBS. After baseline measurements, the sensor was loaded with serial concentrations of antibodies, and both binding and dissociation phases were determined using the Sartorius Octet R8 system (Sartorius, Germany). The data were analyzed using ForteBio data analysis software to determine the binding affinity of the antibodies.

### MUNANA assay

NA inhibition was assessed using the NA-Fluor Influenza Neuraminidase Assay Kit (Applied Biosystems, USA), which measures the ability of F4-Fc to inhibit viral NA-mediated cleavage of a small, soluble fluorescent substrate. The assay was performed following the manufacturer’s instructions. Briefly, serially diluted antibodies were added to white 96-well plates, followed by the addition of diluted virus. After 30 min of incubation at 37°C, 4-MUNANA substrate was added to a final concentration of 200 μM, and the mixture was incubated for 1 h at 37°C. The reaction was terminated by adding 100 µL of NA-Fluor Stop Solution (0.2 M Na_2_CO_3_) to each well. Neuraminidase inhibitory activity was evaluated using a fluorescence-based assay on a BioTek Synergy microplate reader. Fluorescence signals were recorded at an excitation wavelength of 350 nm–365 nm and an emission wavelength of 440 nm–460 nm. The resulting data were processed using GraphPad Prism 9.0 software, and IC50 values were calculated as the antibody concentrations required to achieve 50% inhibition of NA activity compared with the negative control group.

### Enzyme-linked lectin assay (ELLA)

Fetal bovine fetuin (Sigma-Aldrich, Germany) was diluted to 25 μg/mL in coating solution (KPL, USA) to coat 96-well plates (100 μL per well) overnight at 4°C. The plates were washed with phosphate-buffered saline supplemented with 0.05% Tween 20 three times and blocked with 5% bovine serum albumin for 1 h at room temperature. The virus-antibody mixtures (1:1 ratio, 60 μL each) were pre-incubated for 1 h and transferred to fetuin-coated plates for 18 h incubation at 37°C. Plates were washed and incubated with 5 μg/mL HRP-labeled lectin (Sigma-Aldrich, Germany) for 2 h, followed by colorimetric detection at 450 nm using a microplate reader (BioTek, USA).

### ADCC reporter assays

White flat-bottomed 96-well plates with 1 × 10^4^ MDCK cells (100 μL/well) were seeded and incubated overnight at 37°C with 5% CO₂. The next day, the cells were infected with 100 μL of H1N1 PR8 (MOI = 10) for 12 h. A total of 25 μL of Jurkat reporter cells (2.5 × 10^6^ cells/mL) expressing human FcγRIIIa and a luciferase reporter gene (Promega) were added to virus-infected cells along with 25 μL serially diluted antibodies. After incubation for 6 h at 37°C with 5% CO₂, luciferase activity was detected using a luciferase assay kit (Vazyme, China). Fold change was calculated by normalizing the luciferase activity of antibody-treated wells to that of the no-antibody control wells.

### Plaque-forming assay (PFA)

MDCK cells were seeded into six-well plates and incubated overnight. The following day, the cells were washed twice with PBS and infected with serially diluted virus. After 1 h of incubation at 37°C (IAV) or 35°C (IBV) in 5% CO_2_ with gentle rocking every 15 min, the inocula were removed, and cells were overlaid with medium consisting of a 1:1 mixture of 2× DMEM and 1.8% low-melting-point agarose supplemented with 1 μg/mL L-1-tosylamido-2-phenylethyl chloromethyl ketone-treated trypsin (TPCK-treated trypsin). After further incubation for 2–3 days at 37°C (IAV) or 35°C (IBV), cells were fixed with 4% paraformaldehyde for 1 h. The overlays were then removed, and plaques were visualized by staining with 0.5% crystal violet. Viral titers were calculated as PFU per mL.

### *In vivo* protection assay against influenza virus infection

Seven-week-old female BALB/c mice (*n* = 10 per group) were used in this study. For prophylactic evaluation, antibodies were administered via intraperitoneal (i.p.) injection at a dose of 5 mg/kg 24 h prior to intranasal challenge with 5× LD50 of H1N1 (A/Puerto Rico/8/1934), 5× LD50 H3N2 (A/Hong Kong/1/1968), or 5× LD50 Victoria (B/Guangzhou/0215/2012). For the therapeutic setting, mice were challenged first and then treated with antibodies (5 mg/kg, i.p.) 24 h post-infection. At day 4 post-infection, 5 mice of each group were sacrificed. Lung tissues were collected for viral titration via PFA assay or for H&E staining. The scoring of combined pathology was performed according to methods described in previous studies (49, 50). For the remaining five mice in each group, body weight and survival were monitored daily for 14 days. Mice reaching the humane endpoint (≥30% wt loss) were sacrificed and recorded as deaths. Body weight and survival curves were generated using GraphPad Prism (version 9.0).

### Statistical analysis

Statistical analyses were performed using GraphPad Prism version 9 (GraphPad Software, USA). Data are presented as mean ± SD. Differences between treatment and control groups were assessed using a two-tailed Student’s *t*-test (*P < 0.05; **P < 0.01; ***P < 0.001; ****P < 0.0001; ns, not significant).

## Data Availability

All data generated or analyzed during this study are included in this article and its supplemental material. Additional raw data supporting the conclusions of this study are available from the corresponding author upon reasonable request.

## SUPPLEMENTAL MATERIAL

Supplemental material jvi.00874-26-s0001.docx Fig. S1 to S3; Tables S1 to S4.

ASM does not own the copyrights to Supplemental Material that may be linked to, or accessed through, an article. The authors have granted ASM a non-exclusive, world-wide license to publish the Supplemental Material files. Please contact the corresponding author directly for reuse.
