# Supplementary material for: A neuraminidase-targeting nanobody as a therapeutic candidate against influenza A and B viruses
Source: J Virol. 2026 Aug 31;100(9):e00874-26. doi: 10.1128/jvi.00874-26 (PMC13595958; doi:10.1128/jvi.00874-26)
Supplement: Supplementary file 1 [file jvi.00874-26-s0001.docx]

**Supplementary Figure 1**

**Supplementary Figure 1. The expression and purification of recombinant NA protein.** Recombinant NA proteins were expressed in 293F cells, and the expression of proteins was analyzed by SDS-PAGE.

**Supplementary Figure 2**

**Supplementary Figure 2. Enzymatic Activity Assay of the recombinant NA protein.** The enzymatic activity of the NA protein was measured using the NA-Star assay. α2-3,6,8 neuraminidase (New England Biolabs, USA) was used as a positive control.

**Supplementary Figure 3**

**Supplementary Figure 3. Inhibitory efficacy of F4-Fc against swine H1N1 virus.** Virus was pre-incubated with 25 μg/mL antibody and used to infect MDCK monolayers for 48 h. HA titers in the supernatants were measured by hemagglutination assay. 1G01 served as a positive control.

**Supplementary table 1. Identification of Cross-reactive Nanobodies by Binding ELISA**

| **Clones** | **OD450** | | | | |
| --- | --- | --- | --- | --- | --- |
|  | **NA_H1N1** | **NA_H3N2** | **NA_H5N6** | **NA_H7N9** | **NA_H9N2** |
| A2 | 0.073 | 0.075 | 0.095 | 0.145 | 2.082 |
| A3 | 0.08 | 0.144 | 0.089 | 0.139 | 1.75 |
| A4 | 0.181 | 0.116 | 0.087 | 0.083 | 1.525 |
| A7 | 0.189 | 0.111 | 0.076 | 0.119 | 0.856 |
| A11 | 0.138 | 0.176 | 0.105 | 0.141 | 1.366 |
| B2 | 0.158 | 0.121 | 0.083 | 0.088 | 1.312 |
| B6 | 1.227 | 0.583 | 0.643 | 1.053 | 1.707 |
| B7 | 0.153 | 0.129 | 0.079 | 0.111 | 1.7 |
| B8 | 0.251 | 0.145 | 0.085 | 0.125 | 1.507 |
| B9 | 0.176 | 0.076 | 0.053 | 0.106 | 1.747 |
| C1 | 0.19 | 0.09 | 0.074 | 0.062 | 1.43 |
| C7 | 0.197 | 0.119 | 0.147 | 0.158 | 1.614 |
| C8 | 0.274 | 0.144 | 0.083 | 0.181 | 0.626 |
| C10 | 0.243 | 0.146 | 0.092 | 0.155 | 1.741 |
| D1 | 1.229 | 1.076 | 0.728 | 1.095 | 1.416 |
| D5 | 0.208 | 0.116 | 0.088 | 0.073 | 0.693 |
| D7 | 0.232 | 0.135 | 0.075 | 0.124 | 1.415 |
| D8 | 0.257 | 0.15 | 0.076 | 0.146 | 1.397 |
| D9 | 0.082 | 0.167 | 0.054 | 0.158 | 1.243 |
| E2 | 0.278 | 0.056 | 0.086 | 0.055 | 1.918 |
| E8 | 0.26 | 0.089 | 0.092 | 0.172 | 1.552 |
| E12 | 0.102 | 0.132 | 0.087 | 0.088 | 1.015 |
| F1 | 0.159 | 0.122 | 0.063 | 0.214 | 1.806 |
| F4 | 0.243 | 0.074 | 0.079 | 0.082 | 0.692 |
| F5 | 0.25 | 0.186 | 0.08 | 0.141 | 1.689 |
| F7 | 0.341 | 0.512 | 0.348 | 1.094 | 1.11 |
| F12 | 0.315 | 0.07 | 0.088 | 0.086 | 1.193 |
| G5 | 0.114 | 0.114 | 0.081 | 0.074 | 1.26 |
| H1 | 0.162 | 0.563 | 0.506 | 1.285 | 1.617 |
| H2 | 0.279 | 0.086 | 0.085 | 0.087 | 0.742 |
| H4 | 0.577 | 0.512 | 0.303 | 1.307 | 1.484 |
| H10 | 0.778 | 0.509 | 0.36 | 0.759 | 1.313 |

**Supplementary table 2. Identification of Nanobodies Competing with 1G01 for Binding to N2**

| **Clones** | **inhibition%** | **OD450** | |
| --- | --- | --- | --- |
|  |  | **PBS** | **1G01** |
| D1 | 59.87% | 2.213 | 0.888 |
| D7 | 15.74% | 3.691 | 3.11 |
| D8 | 13.85% | 3.719 | 3.204 |
| F4 | 45.49% | 2.385 | 1.3 |
| F5 | 14.85% | 3.144 | 2.677 |
| F7 | 49.29% | 2.942 | 1.492 |
| G2 | 32.52% | 2.838 | 1.915 |
| H1 | 19.22% | 2.857 | 2.308 |
| H4 | 16.09% | 2.473 | 2.073 |

**Supplementary table 3. Half maximal effective concentration (EC50) Values of F4-Fc**

| **Clones** | B6 | D1 | F4 | F7 | H1 | H4 | H10 | 1G01(positive control) | NC-Fc (negtive control） |
| --- | --- | --- | --- | --- | --- | --- | --- | --- | --- |
| **EC50 (nM)** | 0.007 | 0.002 | 1.236 | 0.040 | 0.048 | 0.027 | 0.038 | 0.129 | N.D. |

N.D., not determined because EC50 not reached.

**Supplementary table 4. Half maximal inhibitory concentration (IC50) and Minimal inhibitory concentration (MIC) of antibodies**

| **NA subtype** | **Strain** | **ELLA (μg/mL)** | | | **MUNANA (μg/mL)** | | | **MN (μg/mL)** | | |
| --- | --- | --- | --- | --- | --- | --- | --- | --- | --- | --- |
|  |  | **F4-Fc** | **1G01** | **NC-Fc** | **F4-Fc** | **1G01** | **NC-Fc** | **F4-Fc** | **1G01** | **NC-Fc** |
| N1 | A/Puerto Rico/8/1934 (H1N1) | 0.101 | 0.187 | ＞100 | 0.348 | 0.137 | ＞100 | 0.313 | 1.560 | ＞100 |
| N1 | A/Harbin/HL-1/2024 (H1N1) | 0.038 | 0.205 | ＞100 | 0.273 | 0.487 | ＞100 | 1.560 | 3.200 | ＞100 |
| N2 | A/Hong Kong/1/1968 (H3N2) | 0.558 | 0.003 | ＞100 | 50.400 | 0.089 | ＞100 | ＞100 | 0.313 | ＞100 |
| N2 | A/Guangzhou/086/2022 (H3N2） | ＞100 | 0.094 | ＞100 | ＞100 | 0.217 | ＞100 | ＞100 | 41.667 | ＞100 |
| N2 | A/duck/Hong Kong/Y280/97 (H9N2) | 0.057 | 0.007 | ＞100 | 27.190 | 0.130 | ＞100 | 12.500 | 0.313 | ＞100 |
| B/Victoria/2/87-like | B/Guangzhou/0215/2012 (Victoria) | 0.023 | ＞100 | ＞100 | 6.118 | ＞100 | ＞100 | 5.000 | ＞100 | ＞100 |
| B/Victoria/2/87-like | B/Austria/1359417/2021 (Victoria) | 0.238 | 0.154 | ＞100 | 5.597 | 22.300 | ＞100 | 2.500 | 1.560 | ＞100 |
